# Empirical approach to threshold determination for the delineation of built-up areas with road network data

**DOI:** 10.1371/journal.pone.0194806

**Published:** 2018-03-27

**Authors:** Qi Zhou, Lei Guo

**Affiliations:** 1 School of Information Engineering, China University of Geosciences, Wuhan, P.R. China; 2 Beijing Key Laboratory of Urban Spatial Information Engineering, Beijing, P.R. China; Universidade de Vigo, SPAIN

## Abstract

Various approaches have been proposed to address the delineation of built-up areas for a wide range of applications. Recently developed approaches are based on the increasing availability of road network data. However, most approaches have employed one or more parameters to divide built-up from non-built-up areas. Very few studies have discussed how to determine appropriate thresholds for such parameters. This study employed an empirical approach for threshold determination, and validated that the approach is applicable for the delineation of built-up areas using road network data. A series of experiments were designed to investigate the most-appropriate thresholds (determined using a similarity measure) for multiple parameters of three existing approaches (street blocks, grid-based, and kernel density) with regard to different administrative regions and cities/towns. The results show that in most cases, the most-appropriate thresholds or ranges for different subdivisions are either identical or overlap—thus validating the use of the most-appropriate thresholds to delineate built-up areas for one or multiple small subdivisions and, by inference, for a much larger region.

## Introduction

The delineation of built-up areas, to create polygons that represent built-up areas in a region[1], can be used for applications such as predicting population growth[2], representing urban spatial development[3–4], identifying urban sprawls[5–8] and mapping land-use patterns[9]. Extensive studies have been focused on delineating built-up areas with different source data, including census data[10–12], postcode data[13], remote sensing data[14–19], settlement and buildings data[20–21], “check-in” data (e.g. Flickr or Sina Weibo[22–23]), and even multiple source data[8]. Nowadays, road network data have become increasingly available. Many authoritative mapping agencies have begun to open their data to the public (e.g. TIGER/Line® and Ordnance Survey OpenData), and along with the development of Web 2.0 technology, a number of platforms (e.g. OpenStreetMap and Wikimapia) support volunteers in the creation and distribution of free geographic data for the world. Therefore, the delineation of built-up areas with road network data has received much attention to [1,6,7,24]. For instance, two approaches have been proposed—the grid-based and kernel density approaches—which were derived by analyzing the density of intersections in a road network[24]. The grid-based approach created a regular grid and calculated the number of road intersections in each grid cell; if the density of a grid cell was larger than a certain threshold, such a grid cell was most likely to be a built-up area. This approach has also been improved by first clustering road intersections [6]. The kernel density approach used the kernel density estimation (KDE) to calculate the density of road intersections in each grid cell and delineated built-up areas. A different approach based on street blocks was also proposed [25], and its steps were to first calculate the land areas (or sizes) of all the street blocks in a road network, and then to divide these street blocks into built-up areas and non-built-up areas.

Nevertheless, existing approaches involve one or more parameter(s) for the delineation of built-up areas with road network data. For instances, the approach based on street blocks involves at least one parameter called the street block size; both the grid-based and kernel density approaches involve two parameters—cell size and cell density and bandwidth and kernel density, respectively[1]. However, the thresholds for the above parameters were determined arbitrarily and subjectively in previous studies. Some suggested setting the bandwidth between 250 and 500 m and the cell size at 100 m [24]; whereas some others used a threshold of 500 m for the cell size [6]. In another study, the appropriate thresholds for these parameters were determined through visual analysis rather than quantitative assessment[1]. Threshold determination is a necessary step, especially for automatic delineation of a large number of built-up areas in a country/region. To our knowledge, very few studies have quantitatively analyzed the most-appropriate thresholds for various parameters; and more importantly, how to determine appropriate threshold(s) for the automatic delineation of a large number of built-up areas with road network data. While a threshold determination approach called the head/tail break (dividing many small things and a few large things according to the arithmetic mean) has been proposed [25], there is a need to determine the most-appropriate threshold from multiple breaks obtained using the head/tail break approach. An empirical approach was also proposed [26], and the tenet of it was to divide a large road network into several subdivisions, and to use the most-appropriate threshold(s), obtained from one or several subdivisions, to make inferences for large road networks. But, the empirical approach has only been used for selective omission in a road network data (i.e. retaining more important roads for the purpose of map generalization) and not for the delineation of built-up areas with road network data.

This study was inspired by the empirical approach [26]. The objective of this study is to validate whether or not the empirical approach is also applicable to the delineation of built-up areas with road network data. More specifically, we assumed that the most-appropriate thresholds/ranges are either the same or overlap, for the delineation of built-up areas in the different subdivisions of a road network. If this assumption is true, it is possible to use the most-appropriate thresholds obtained from one or multiple small subdivisions to make inferences for a much larger region. This study used five parameters of three typical approaches (the approach based on street blocks, the grid-based approach, and the kernel density approach) for analyses; and a similarity measure was used to determine the most-appropriate thresholds for various parameters. However, a comparison of these approaches, having already been reported in a previous research work [1], is beyond the scope of this study.

The paper is structured as follows: Section 2 describes the experimental data, benchmarks, approaches, and parameters to be tested for the delineation of built-up areas; and the measure used to determine and compare multiple appropriate thresholds. Section 3 analyzes the most-appropriate thresholds for various parameters. Section 4 further validates the results of using a different benchmark, evaluation measure and/or study area. Section 5 presents conclusion and discussions.

## Experimental design

### Data and benchmark

**Fig 1:**
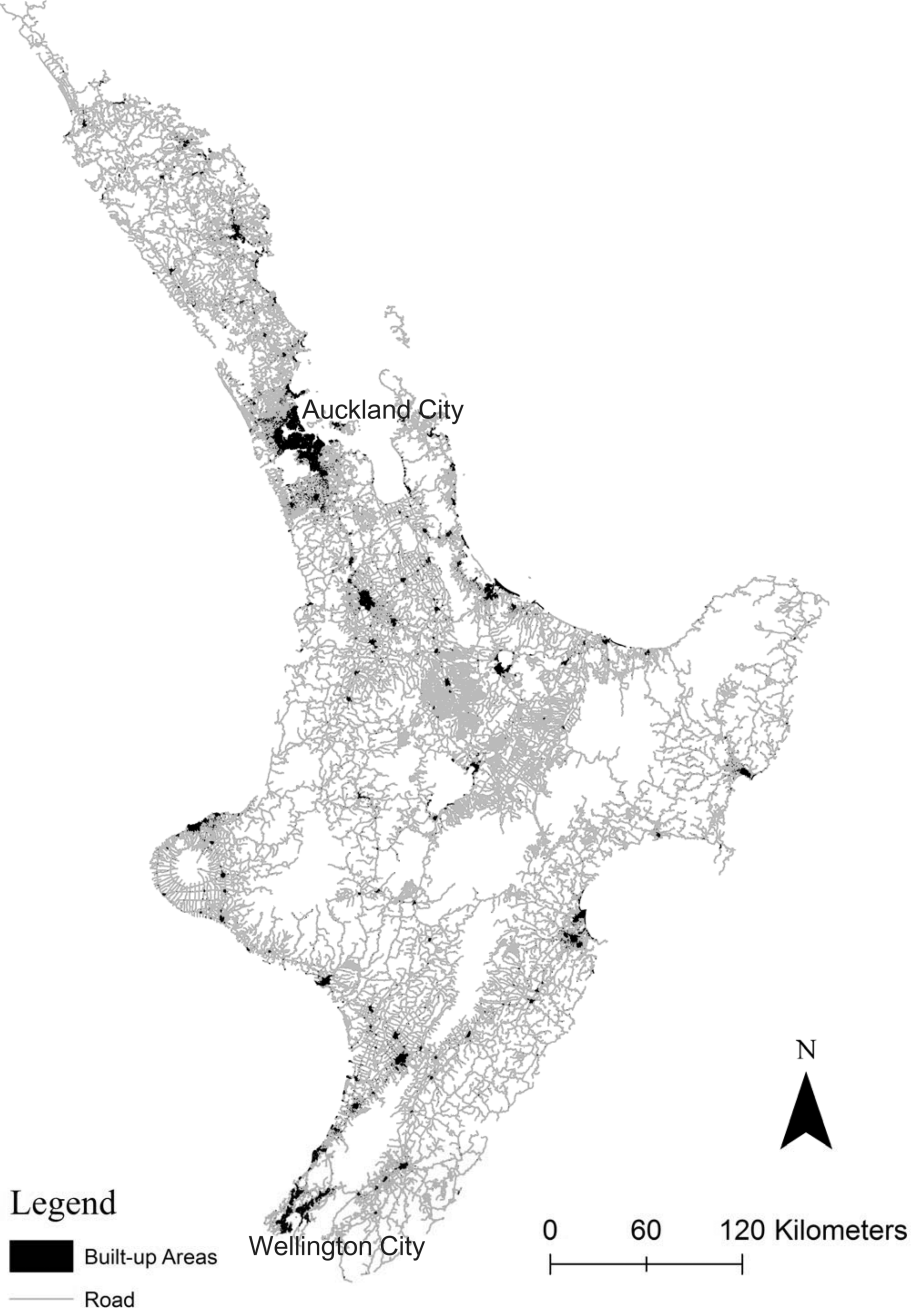
Study area: North Island of New Zealand.

This study used the open data produced by the Land Information of New Zealand (http://www.linz.govt.nz/topography/topo-maps/map-chooser) for testing, see S1 File. More precisely, the road network data at 1: 50,000 scale were used as source data, and the corresponding building and residential data at 1: 250,000 scale were used as benchmarks (“Fig 1”). The building and residential data are described by the Land Information of New Zealand as "central business district areas of large towns and cities", and "a group of houses and buildings that covers an area greater than 90,000 m^2^", respectively. These benchmarks were chosen because they have also been used for evaluating the automatic delineation of built-up areas in previous studies[1,20].

### Approaches and parameters to be tested

Several approaches have been proposed for the delineation of built-up areas using road network data. This section briefly introduces the three typical approaches—the approach based on street blocks, the grid-based approach, and the kernel density approach—that will be tested. More details are available in previous research work [1,24,25].

#### The approach based on street blocks

**Fig 2:**
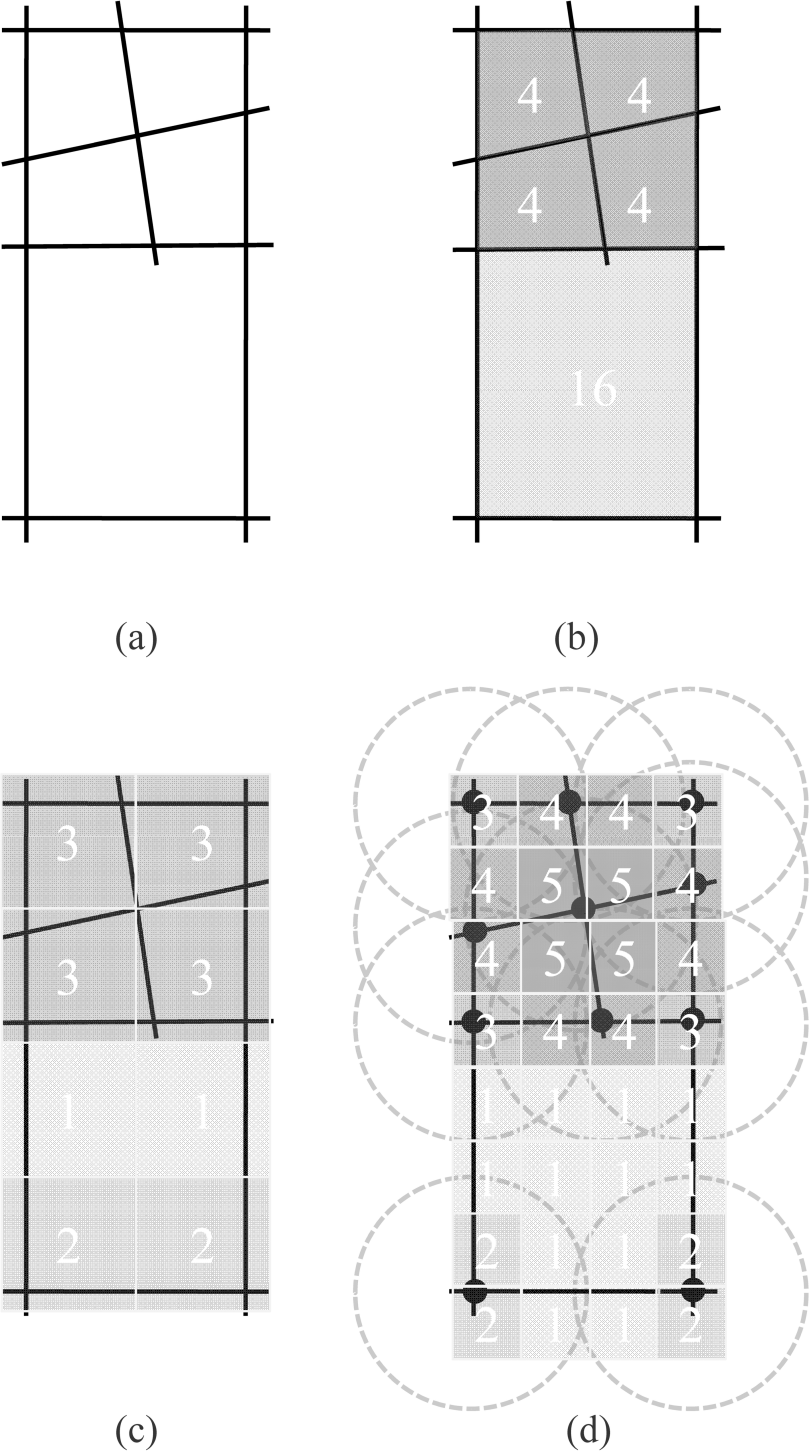
Three approaches for the delineation of built-up areas: (a) a schematic road network, (b) the approach based on street blocks, (c) the grid-based approach, and (d) the kernel density approach.

A street block can be viewed as a closed region formed by one or several connected roads [1]. The approach based on street blocks has two general steps [25]:

1. Calculate the land areas of all the street blocks (e.g., “4” and “16” in “Fig 2B”) in a road network data.
2. Delineate the street blocks whose land areas are smaller than a threshold (called the street block size) as built-up areas (e.g., “4”in “Fig 2B”).

#### The grid-based approach

The grid-based approach may have four steps [24]:

1. Create a regular grid (e.g. 0.5×0.5 km^2^);
2. Intersect this grid with road network data;
3. Calculate the density (*D*_*i*_) of each grid cell according to the formula below[1]:
  $$D_{i}=\frac{L_{i}}{A_{i}}$$ (1)
  where *L*_*i*_ denotes the total length of roads located in the *i*^th^ grid cell; and *A*_*i*_ denotes the area of the *i*^th^ grid cell.
4. Delineate the grid cells whose densities are larger than a threshold (called the grid density) as built-up areas (e.g. “3”in Fig 2C).

The grid-based approach involves two parameters. In addition to the cell density, the cell size is also an essential parameter because the cell density may vary with different cell sizes.

#### The kernel density approach

The kernel density approach may have two general steps [24]:

1. Calculate the density of each cell (e.g. “1”, “2”, “3”, “4”, and “5” in “Fig 2D”) using the kernel density estimation:
  $$\overset{}{\hat{\lambda }}(s)=\sum_{i=1}^{n}\frac{1}{\tau^{2}}k\left(\frac{s-s_{i}}{\tau }\right)$$ (2)
  where $\hat{\lambda }(s)$ is the estimated density of the cell measured at location *s* (i.e. the centroid of this cell), τ is the bandwidth, *n* is the number of neighboring road intersections of location *s*; and *s*_*i*_ is the *i*^th^ road intersection within distance τ of location *s*; *k* (…) is the kernel function.
2. Delineate the grid cells whose kernel densities are larger than a threshold (called the kernel density) as built-up areas (e.g. “3” and larger values in “Fig 2D”).

The kernel density approach also involves two parameters—the bandwidth (the radius of the dot circles in “Fig 2D”), and the kernel density. With the bandwidth, the cell size may be set as small as possible. However, if the cell size is set too small, the number of cells increases dramatically and the approach may fail due to the ''out of memory'' problem [1]. Thus, by using this approach, we fixed the cell size at 0.1×0.1 km^2^.

### Experimental steps

An experiment was designed to investigate whether the most-appropriate thresholds for the delineation of built-up areas are the same or similar for different subdivisions of a road network. Specifically:

1. Divide a large road network into subdivisions.
2. Determine the most-appropriate thresholds for the delineation of built-up areas for each subdivision.
3. Compare the most-appropriate thresholds for different subdivisions.

#### Divide a large road network into subdivisions

**Fig 3:**
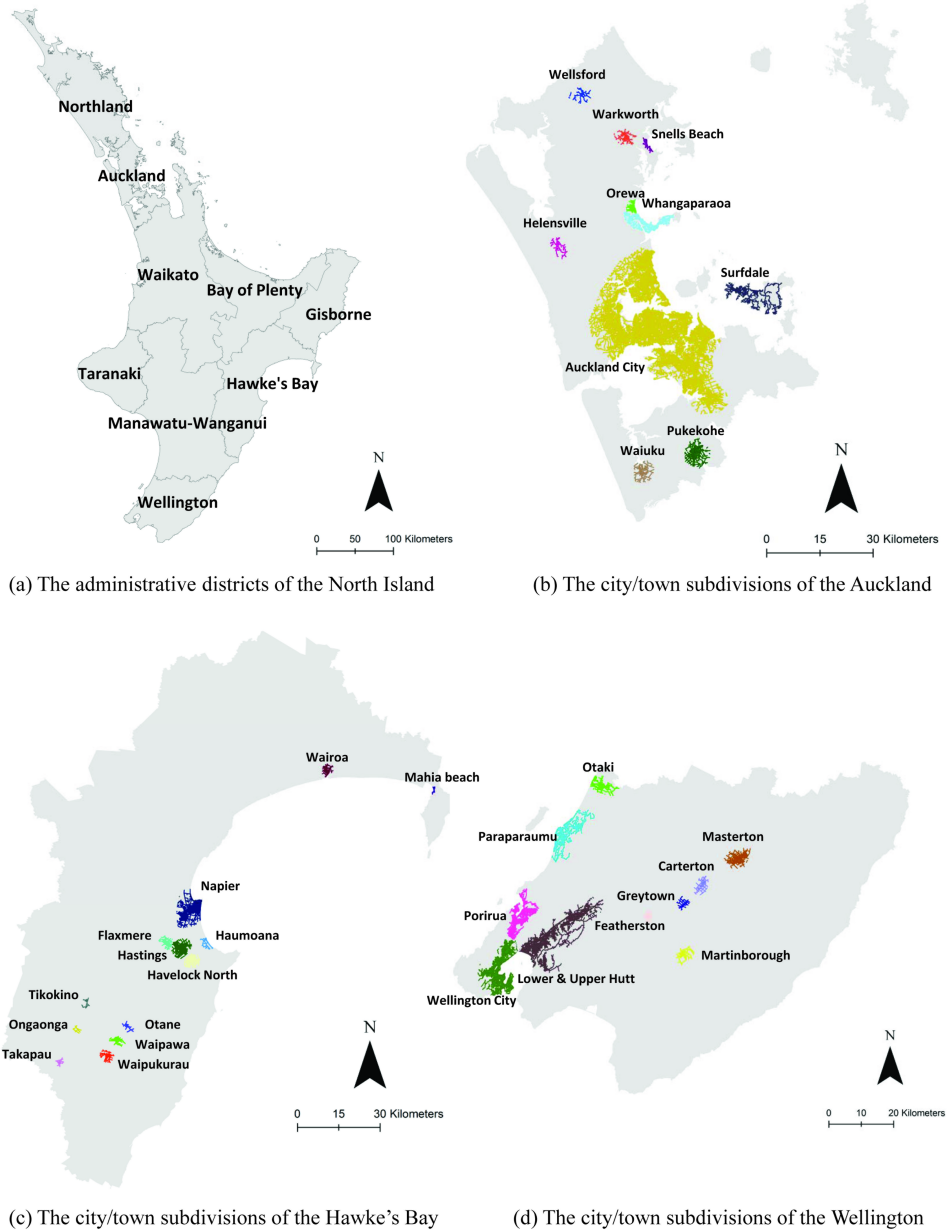
Subdividing the road network of the North Island according to (a) nine administrative districts and (b–d) different cities or towns.

Two division modes were considered:

1. The road network of the North Island was subdivided into nine non-overlapping road networks using the administrative boundary data in order to investigate the most-appropriate thresholds for the different administrative districts (“Fig 3A”).
2. The road network of the North Island was also subdivided into smaller road networks in order to investigate the most-appropriate thresholds for cities or towns of different sizes. As an example, a total of 33 subdivisions in three (Auckland, Wellington and Hawke’s Bay) out of the nine administrative districts were manually chosen (“Fig 3B”, “Fig 3C,” “Fig 3D”) by referring to the actual built-up areas in the corresponding benchmark. The rules for selection include: first, each subdivision covers the built-up areas in the benchmark of only one city or town, but this subdivision should be larger than the corresponding built-up areas in the benchmark; and second, the selected built-up areas in different subdivisions should vary dramatically in size. Specifically, the size of the largest built-up area (Auckland City) among the 33 subdivisions is 302.627 km^2^, while that of the smallest one (Tikokino) is only 0.169 km^2^.

#### Determine the most-appropriate threshold for each subdivision

The basic idea is to first automatically delineate built-up areas with different thresholds. The delineated built-up areas are then compared with those in the corresponding benchmark by calculating a similarity measure. Finally, the threshold corresponding to the highest similarity value is determined as the most-appropriate threshold. The similarity value, ranging from 0 to 1, can be calculated as [26,27]:

$$Similarity=\frac{A\cap B}{A+B-A\cap B}$$ (3)

where *A* is the land area of the automatically delineated built-up areas; *B* is the land area of the built-up areas in the corresponding benchmark; and *A* ∩ *B* is the land area of the built-up areas delineated in common.

**Fig 4:**
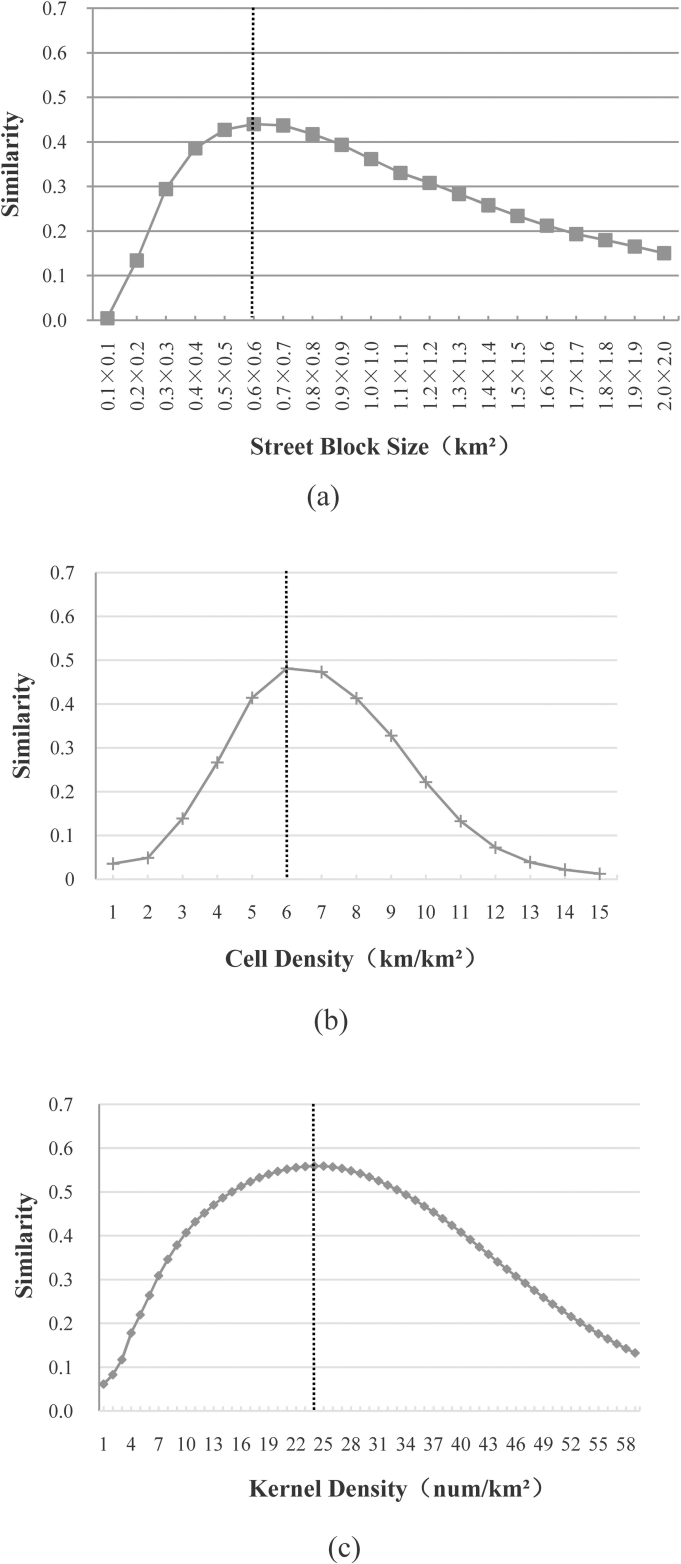
Most-appropriate thresholds for the North Island of New Zealand using (a) the approach based on street blocks; (b) the grid-based approach; (c) the kernel density approach.

“Fig 4” plots the similarity distributions for the North Island of New Zealand, using three approaches for the delineation of built-up areas. The x-axis denotes various thresholds for one parameter (e.g., street block size, cell density or kernel density), and the y-axis denotes the corresponding similarity value. In this study, these three approaches were implemented using commercial GIS software (ArcGIS, version 9.3) and freeware (OpenJUMP, version 1.6.0). The details steps to implement these approaches have been reported in a previous study [1]. It can be seen in “Fig 4” that all the similarity distributions have the same trend. That is, the similarity value first increases along with an increase of the threshold, and then it begins to decrease after going through a peak. The threshold corresponding to this peak can be viewed as the most-appropriate threshold (highlighted with a vertical dotted line). More precisely, the most-appropriate threshold for the street block size is 0.6×0.6 (km^2^), while those for the cell density and the kernel density are 6 (km/km^2^) and 24 (num/km^2^), respectively. Based on the similarity distributions in “Fig 4”, the parameters and thresholds to be tested in this study are listed in “Table 1”.

**Table 1:** Parameters and thresholds to be tested using three approaches to the delineation of built-up areas.

	Parameter	Thresholds
**Approach based on street blocks**	Street block size (km^2^)	0.1×0.1–2.0×2.0
**Grid-based approach**	Cell size (km^2^)	0.3×0.3; 0.5×0.5; 0.7×0.7; 0.9×0.9
	Cell density (km/km^2^)	1–15
**Kernel density approach**	Bandwidth (km)	0.3; 0.5; 0.7; 0.9
	Kernel density (num/km^2^)	1–60

#### Compare the most-appropriate thresholds for different subdivisions

The most-appropriate thresholds for the nine administrative districts and 33 test regions covering cities or towns were determined. Initially, the most-appropriate thresholds were compared to find out if they were identical. If the most-appropriate thresholds turned out not to be identical, the appropriate threshold ranges were visually determined to investigate whether the threshold ranges overlapped.

The head/tail break, as an existing classification method, was also employed for comparison purposes. The head/tail break generally applies a break rule for threshold determination. The break rule is defined as “Given a variable X, if its values x follow a heavy-tailed distribution, then the mean of the values can divide all the values into two parts: a high percentage in the tail, and a low percentage in the head.”[25]. It has been found that all the street block sizes of a road network follow the heavy-tailed distribution. The mean size was then used as a threshold to divide all the street blocks into built-up areas (in the tail) and non-built-up areas (in the head) [25]. When the first mean is not perfect, the break may continue if all the street block sizes in the tail still follow the heavy-tailed distribution. Thereby, the second mean, the third mean, the fourth mean and so on may be obtained. The break stops if the percentage in the head is larger than 40% [28]. This condition can be relaxed by 50%, or even more if the percentage in the head remained less than 40% in a subsequent break. This study also employs the head/tail break method to determine thresholds for the street block size, and also for the cell density and kernel density; these thresholds are also evaluated using the similarity measure.

## Experimental results and analyses

### Results of the similarity distributions for the nine administrative districts of the North Island

#### Results for the approach based on street blocks

**Fig 5:**
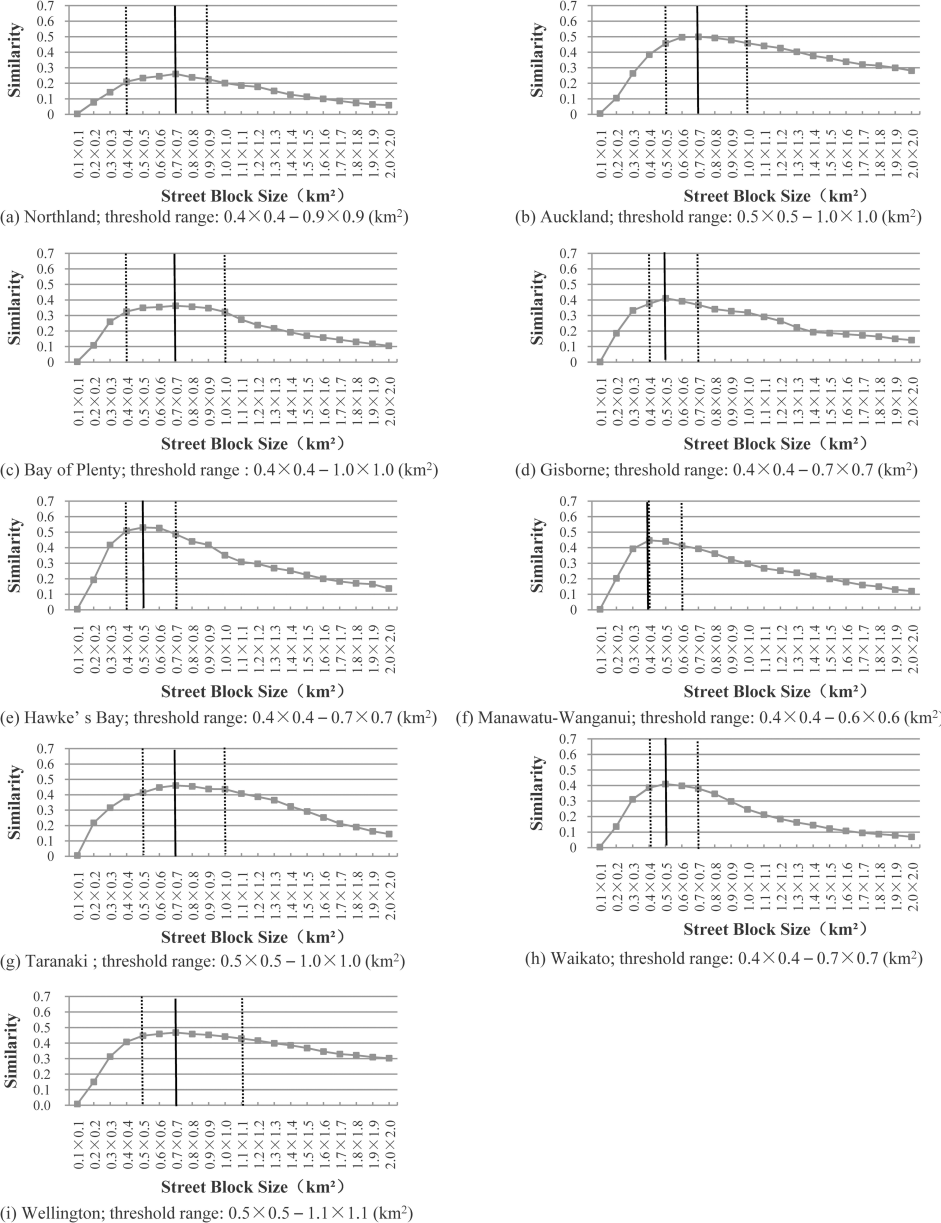
Most-appropriate thresholds for the nine administrative districts of the North Island, using the approach based on street blocks. The most-appropriate threshold for each administrative district is highlighted with a vertical solid line.

“Fig 5” plots the similarity distributions for the nine administrative districts of the North Island, using the approach based on street blocks. The threshold for the only parameter (street block size) varies from 0.1×0.1 to 2.0×2.0 km^2^. For each administrative district, the most-appropriate threshold for the street block size is highlighted with a vertical, solid line.

The following can be observed from “Fig 5”:

1. All the distributions are similar to those plotted in “Fig 4A”. That is, the similarity value first increases with an increase of the street block size, and then it begins to decrease after going through a peak.
2. The most-appropriate thresholds for the street block size are all located within 0.4×0.4 to 0.7×0.7km^2^. To be specific, the most-appropriate thresholds for five out of the nine administrative districts are 0.7×0.7km^2^, while those for three out of the nine administrative districts are 0.5×0.5km^2^.
3. An appropriate threshold range (highlighted with two vertical dotted lines), in which all the corresponding similarity values are no more than 0.05 different from that of the most-appropriate threshold, was determined for each administrative district. For instance, the appropriate threshold range for Northland is between 0.4×0.4 and 0.9×0.9 km^2^, while that of Auckland is 0.5×0.5 to 1.0×1.0 km^2^. More importantly, all the appropriate threshold ranges overlap in the nine administrative districts.

#### Results for the grid-based approach

**Fig 6:**
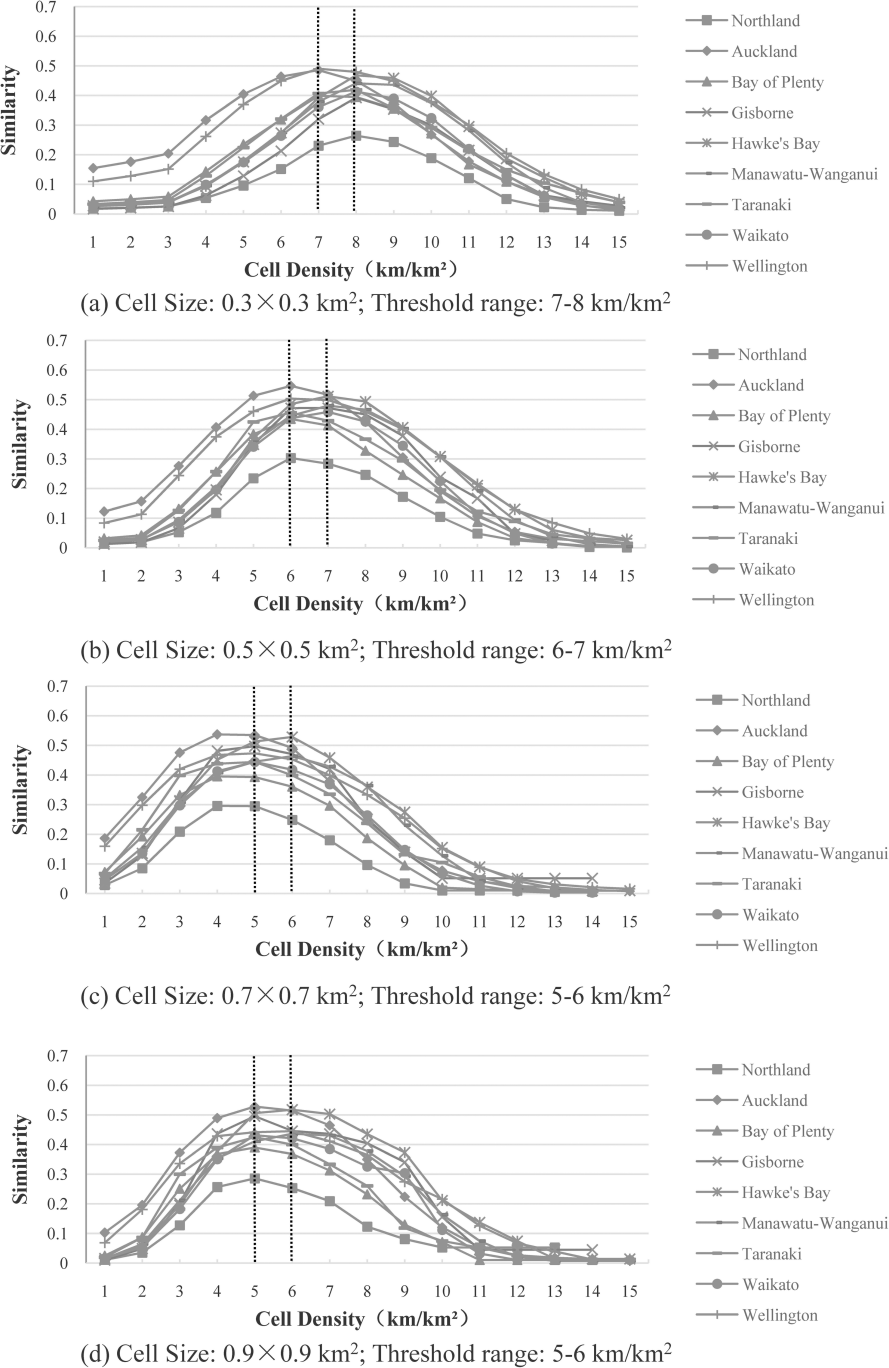
Most-appropriate thresholds for the nine administrative districts of the North Island, using the grid-based approach with different cell sizes: (a) 0.3×0.3 km^2^, (b) 0.5×0.5 km^2^, (c) 0.7×0.7 km^2^, and (d) 0.9×0.9 km^2^. For each cell size, the most-appropriate threshold range is highlighted with two vertical dotted lines.

“Fig 6” plots the similarity distributions for the nine administrative districts of the North Island using the grid-based approach. The grid-based approach involves two parameters: cell size (ranging from 0.3×0.3 to 0.9×0.9 km^2^), and cell density (ranging from 1 to 15 km/km^2^).

The following can be observed from “Fig 6”:

1. All the distributions are similar to those plotted in “Fig 4B”, despite the use of different cell sizes (i.e. 0.3×0.3 to 0.9×0.9 km^2^) and different cell densities (i.e., 1–15 km/km^2^).
2. For each cell size, the most-appropriate thresholds for the cell density are either the same or only one apart (e.g., 6–7 km/km^2^ for the cell size 0.5×0.5 km^2^).

The maximum similarity values for the different cell sizes were compared (“Table 2”). It can be seen in “Table 2” that the most-appropriate cell size appears to be 0.5×0.5 km^2^, for which the maximum similarity value becomes the greatest for seven out of the nine administrative districts.

**Table 2:** Maximum similarity values for different cell sizes obtained from the nine administrative districts of the North Island.

	Maximum similarity for different cell sizes (km^2^)			
	0.3×0.3	0.5×0.5	0.7×0.7	0.9×0.9
**Northland**	0.264	**0.303**	0.295	0.285
**Auckland**	0.485	**0.546**	0.537	0.528
**Bay of Plenty**	0.399	**0.434**	0.395	0.389
**Gisborne**	0.392	0.472	**0.497**	0.496
**Hawke’s Bay**	0.468	0.511	**0.528**	0.517
**Manawatu-Wanganui**	0.441	**0.479**	0.463	0.437
**Taranaki**	0.419	**0.456**	0.444	0.425
**Waikato**	0.413	**0.458**	0.444	0.431
**Wellington**	0.490	**0.504**	0.473	0.445

#### Results for the kernel density approach

**Fig 7:**
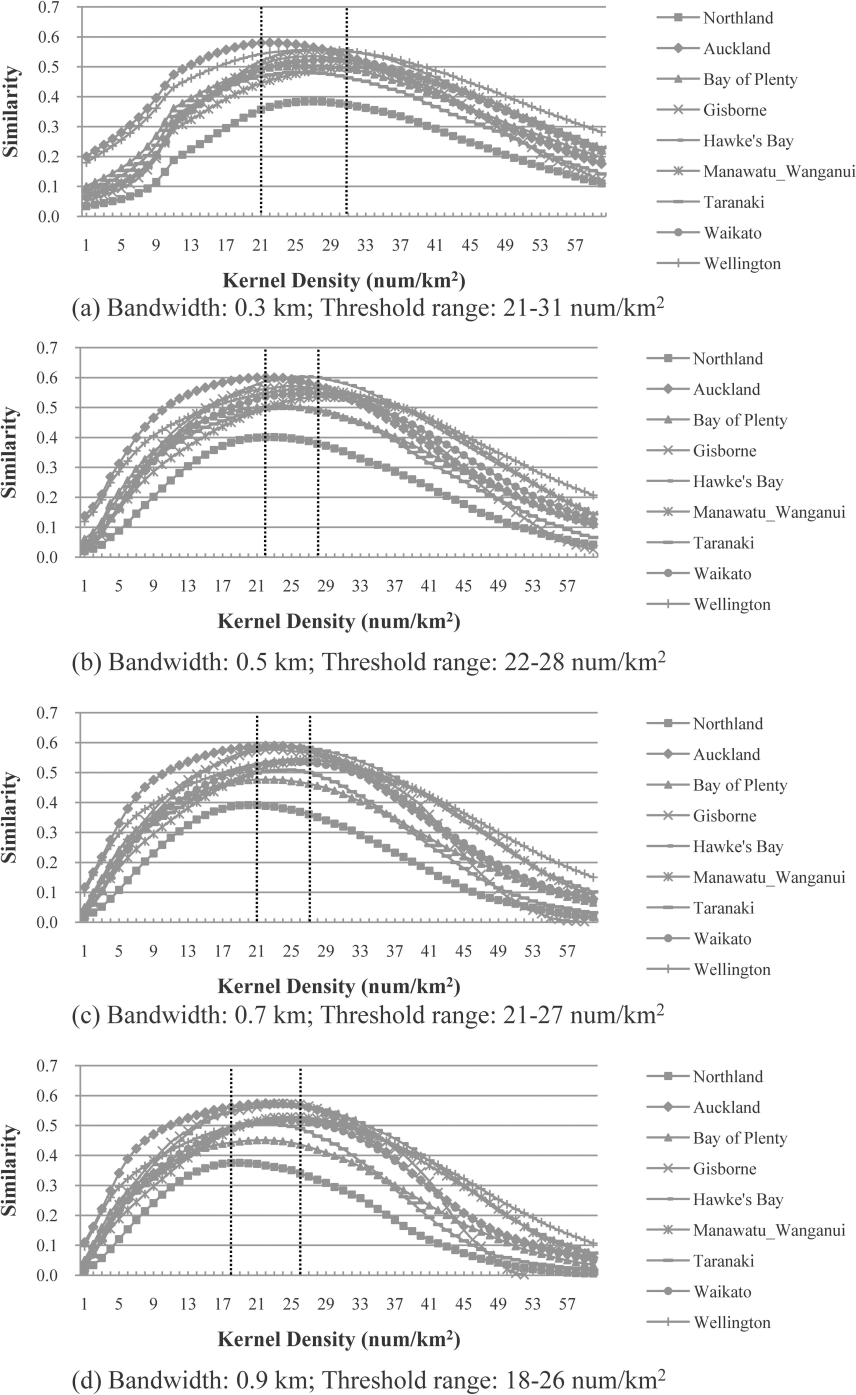
Most-appropriate thresholds for the nine administrative districts of the North Island, using the kernel density approach with different bandwidths: (a) 0.3 km, (b) 0.5 km, (c) 0.7 km, and (d) 0.9 km. For each bandwidth, the most-appropriate threshold range is highlighted with two vertical dotted lines.

“Fig 7” plots the similarity distributions for the nine administrative districts of North Island using the kernel density approach. The two parameters, bandwidth (ranging from 0.3 to 0.9 km), and kernel density (ranging from 1 to 60 num/km^2^) in the kernel density approach were analyzed.

The following can be observed from “Fig 7”:

1. The most-appropriate thresholds are located within a certain range (e.g., 22–28 num/km^2^ for the bandwidth 0.5 km), in which the similarity values are either the same or close to each other.
2. The ranges for the different bandwidths (i.e., 0.3, 0.5, 0.7 and 0.9 km) overlap.

“Table 3” further lists the maximum similarity values for different bandwidths, tested on the nine administrative districts of the North Island. It can be seen in “Table 3” that the most-appropriate bandwidth is 0.5 km for six out of the nine administrative districts and 0.7 km for another three districts.

**Table 3:** Maximum similarity values for different bandwidths obtained from the nine administrative districts of the North Island.

	Maximum similarity for different bandwidths (km)			
	0.3	0.5	0.7	0.9
**Northland**	0.384	**0.401**	0.392	0.375
**Auckland**	0.580	**0.601**	0.589	0.574
**Bay of Plenty**	0.505	**0.505**	0.476	0.451
**Gisborne**	0.536	0.573	**0.578**	0.575
**Hawke’s Bay**	0.559	**0.603**	0.588	0.565
**Manawatu-Wanganui**	0.498	0.535	**0.542**	0.529
**Taranaki**	0.482	0.501	**0.509**	0.508
**Waikato**	0.523	**0.547**	0.536	0.517
**Wellington**	0.557	**0.563**	0.545	0.524

### Results of the similarity distributions for 33 cities/towns on the North Island

“Table 4” lists the most-appropriate thresholds or ranges for the parameters (i.e., street block size, cell density and kernel density) tested on the 33 subdivisions of the North Island, also see S2 File. The threshold for the cell size was fixed at 0.5×0.5 km^2^ while the bandwidth was fixed at 0.5 km, the value at which the corresponding similarity value becomes a maximum in most cases (“Tables 2 and 3”).

**Table 4:** Most-appropriate thresholds or ranges for the 33 subdivisions of the North Island.

No. of Subdivision	Name of built-up areas	Size of built-up areas (km^2^)	Most-appropriate threshold or range		
			Street block size (km^2^)	Cell density (km/km^2^)	Kernel density (num/km^2^)
1	Auckland City	302.627	0.9×0.9	6	20
2	Lower & Upper Hutt	41.676	0.8×0.8	6	27
3	Wellington	38.393	0.7×0.7	7	32
4	Napier	19.693	0.6×0.6	7	30
5	Porirua	18.344	1.3×1.3–1.7×1.7	6	29
6	Paraparaumu	16.502	0.9×0.9–1.0×1.0	5	26
7	Hastings	12.127	0.4×0.4	6	22
8	Whangaparaoa	11.057	0.9×0.9	6	18
9	Masterton	9.238	0.8×0.8	6	23
10	Havelock North	5.543	0.9×0.9	6	25
11	Pukekohe	5.436	0.6×0.6	6	31
12	Surfdale	3.600	0.5×0.5	5	18
13	Orewa	3.156	0.6×0.6–1.7×1.7	6	26
14	Otaki	3.113	0.9×0.9–1.1×1.1	5	25
15	Waiuku	2.837	0.5×0.5	5	24
16	Flaxmere	2.819	0.6×0.6	6	23
17	Carterton	2.389	0.5×0.5	6	21
18	Waipukurau	2.101	0.6×0.6	5	31
19	Wairoa	2.006	0.4×0.4	8	26
20	Snells Beach	1.636	0.4×0.4–+∞	6	17
21	Warkworth	1.598	0.9×0.9–1.1×1.1	6	23
22	Martinborough	1.369	0.4×0.4	8	22
23	Greytown	1.359	0.4×0.4–0.8×0.8	4	14
24	Featherston	1.333	0.3×0.3–0.4×0.4	7	28
25	Helensville	1.089	0.5×0.5–1.6×1.6	6	27
26	Waipawa	1.043	0.7×0.7–1.6×1.6	5	24
27	Wellsford	0.894	0.7×0.7	4	10
28	Haumoana	0.561	1.1×1.1–1.4×1.4	4	16
29	Mahia beach	0.479	0.5×0.5–+∞	5	20
30	Otane	0.316	0.2×0.2	6	30
31	Ongaonga	0.199	0.2×0.2–0.9×0.9	6	12
32	Takapau	0.189	0.2×0.2–0.3×0.3	5–7	28
33	Tikokino	0.169	0.3×0.3–0.4×0.4	5–6	13

The following can be observed from “Table 4”:

1. There may be multiple, most-appropriate thresholds, especially for the subdivisions (e.g., Orewa and Waipawa) with relatively small built-up areas. This is because, typically, the smaller the size of a city or town, the smaller the number of street blocks or grids to be delineated as built-up areas. It is therefore possible to delineate the same built-up areas with different thresholds.
2. For most of the subdivisions, the most-appropriate thresholds for a parameter (e.g., street block size, cell density or kernel density) are either the same or close to each other. For instance, in 29 out of the 33 subdivisions, the most-appropriate thresholds or ranges for the street block size are either located within or overlap with 0.4×0.4 to 0.9×0.9 km^2^, which also overlaps with those found in “Fig 5”. In 28 out of the 33 subdivisions, the most-appropriate thresholds for the cell density are located within 5 to 7 km/km^2^, which is almost the same as the value (6 to 7 km/km^2^) found in “Fig 6B”. In 26 out of the 33 subdivisions, the most-appropriate thresholds for the kernel density are located within 18 to 31 num/km^2^. These thresholds are either within, or close to, the values (22 to 28 num/km^2^) found in “Fig 7B”.
3. However, the ranges of the above most-appropriate thresholds become wider. For instance, the range for the street block size varies from 0.2×0.2 to 1.7×1.7 km^2^. The corresponding result for the cell density varies from 4 to 8 km/km^2^ and for the kernel density, it varies from 10 to 32 num/km^2^. This illustrates that the most-appropriate thresholds for multiple parameters may vary dramatically for cities or towns.

### Results of the head/tail break

“Tables 5–7” list the thresholds obtained using the head/tail break and corresponding similarity values for the approach based on street blocks, the grid-based approach and the kernel density approach, respectively. The thresholds for some parameters (e.g., cell size and bandwidth) and the 33 cities/towns of the North Island are not listed here because their values cannot follow the heavy-tailed distribution.

**Table 5:** Thresholds for the street block size obtained using the head/tail break and the corresponding similarity values for the approach based on street blocks.

	1st Mean	Similarity	2nd mean	Similarity	3rd mean	Similarity
**North Island**	2.26×2.26	0.123	0.57×0.57	0.438	0.23×0.23	0.205
**Northland**	3.08×3.08	0.024	1.14×1.14	0.186	0.37×0.37	0.189
**Auckland**	0.83×0.83	0.489	0.28×0.28	0.239	0.17×0.17	0.063
**Bay of Plenty**	1.80×1.80	0.128	0.53×0.53	0.354	0.23×0.23	0.174
**Gisborne**	2.02×2.02	0.139	0.47×0.47	0.413	0.21×0.21	0.220
**Hawke’s Bay**	2.04×2.04	0.128	0.54×0.54	0.537	0.22×0.22	0.274
**Manawatu-Wanganui**	2.12×2.12	0.109	0.52×0.52	0.434	0.22×0.22	0.253
**Taranaki**	2.05×2.05	0.131	0.52×0.52	0.412	0.21×0.21	0.232
**Waikato**	2.17×2.17	0.058	0.72×0.72	0.373	0.26×0.26	0.275
**Wellington**	1.08×1. 08	0.429	0.27×0.27	0.283	0.16×0.16	0.077

**Table 6:** Thresholds for the cell density obtained using the head/tail break and the corresponding similarity values for the grid-based approach.

	1st mean	Similarity	2nd mean	Similarity	3rd mean	Similarity	4th mean	Similarity	5th mean	Similarity
**North Island**	2.20*	0.066	3.59	0.205	5.68	0.469	8.51*	0.370	10.57*	0.170
**Northland**	2.03*	0.019	2.98	0.052	4.10	0.129	5.42	0.269	7.23	0.281
**Auckland**	2.96	0.272	5.68	0.541	8.59*	0.362	10.54	0.136	12.48	0.041
**Bay of Plenty**	2.15*	0.057	3.39	0.171	5.22	0.399	7.81*	0.352	9.66*	0.190
**Gisborne**	1.96*	0.018	2.82	0.056	4.01	0.183	5.73	0.456	8.47*	0.394
**Hawke’s Bay**	2.09*	0.032	3.19	0.100	4.73	0.322	7.27*	0.512	9.89*	0.314
**Manawatu** **-Wanganui**	2.03*	0.029	3.02	0.096	4.64	0.310	7.26	0.479	9.88*	0.316
**Taranaki**	2.01*	0.038	2.98	0.124	4.62	0.382	7.43*	0.404	9.89*	0.205
**Waikato**	2.09*	0.034	3.19	0.101	4.69	0.310	7.04	0.457	9.29	0.307
**Wellington**	2.67	0.204	5.38	0.485	8.96*	0.404	11.31	0.180	13.32	0.069

*Violates the 40% condition.

**Table 7:** Thresholds for the kernel density obtained using the head/tail break and the corresponding similarity values for the kernel density approach.

	1st mean	Similarity	2nd mean	Similarity	3rd mean	Similarity	4th mean	Similarity	5th mean	Similarity
**North Island**	4.7	0.220	18.5	0.537	39.4*	0.416	55.0	0.176	70.0	0.061
**Northland**	3.1	0.04	7.7	0.164	17.2	0.374	31.0	0.354	44.4*	0.185
**Auckland**	8.5	0.449	30.7*	0.543	47.9	0.252	63.1	0.091	80.1	0.033
**Bay of Plenty**	4.7	0.208	18.8	0.491	38.4*	0.383	53.4	0.174	66.4	0.070
**Gisborne**	2.9	0.070	8.1	0.304	23.3	0.569	39.5*	0.415	48.6*	0.205
**Hawke’s Bay**	3.6	0.094	11.7	0.420	31.5*	0.577	49.0*	0.310	62.6	0.122
**Manawatu** **-Wanganui**	4.0	0.130	15.2	0.414	34.7*	0.519	50.5*	0.291	62.6	0.120
**Taranaki**	4.0	0.163	15.9	0.444	33.2*	0.445	46.0*	0.236	57.0	0.093
**Waikato**	3.7	0.112	13.0	0.421	33.0*	0.515	48.6*	0.277	60.8	0.111
**Wellington**	8.6	0.391	34.0*	0.535	54.9	0.265	74.2	0.095	97.0	0.033

*Violates the 40% condition.

The following can be observed from “Tables 5–7”:

1. The values of both the street block size and kernel density follow the heavy-tailed distribution for the ten study cases. Values of cell density also follow the heavy-tailed distribution, in most study cases, if the relaxation condition is considered (i.e., the percentage in the head is allowed to be larger than 40%).
2. Most of the maximum similarity values obtained using the head/tail break (highlighted with gray) are similar (i.e., with a difference no more than 0.05) to those in “Table 8”. However, the number of breaks needed for a maximum similarity value may vary between study cases. As an example, in “Table 7”, the second mean is the most-appropriate threshold for the study case of Auckland, but the fifth mean is the most-appropriate threshold for the study case of Northland.
3. The mean for the maximum similarity value is either located within or close to the appropriate range. (e.g., 0.4×0.4 to 0.9×0.9 km^2^ for the street block size, 5–7 km/km^2^ for the cell density, and 18–31num/km^2^ for the kernel density) found in “Table 4”. The similarity value may, however, be much lower if the threshold obtained using the head/tail break is outside of the appropriate range (as is seen in the study case of Northland in “Table 5”). Empirical threshold ranges may therefore also be used to determine an appropriate mean for the head/tail break.

**Table 8:** Maximum similarity values of using different approaches for the delineation of built-up areas with road network data.

	Size of built-up areas (km^2^)	Maximum Similarity Value		
		Street block size (km^2^)	Cell density (km/km^2^)	Kernel density (num/km^2^)
**North Island**	937.183	0.440	0.481	0.560
**Northland**	49.925	0.260	0.303	0.401
**Auckland**	350.913	0.496	0.546	0.601
**Bay of Plenty**	82.181	0.354	0.414	0.505
**Gisborne**	15.575	0.411	0.472	0.573
**Hawke’s Bay**	51.182	0.530	0.511	0.603
**Manawatu-Wanganui**	77.287	0.447	0.479	0.535
**Taranaki**	40.766	0.461	0.456	0.501
**Waikato**	133.341	0.409	0.458	0.547
**Wellington**	136.013	0.469	0.504	0.563

## Validation on using a different benchmark, evaluation measure and study area

### Using a different benchmark and evaluation measure

In the previous tests, the benchmark for evaluation consisted of building and residential data only. However, the actual built-up areas may include not only these features, but also roads, school playing fields, and factories. Therefore, the actual built-up areas may be larger than those marked in the benchmarks.

An investigation was made into whether the most-appropriate thresholds or ranges may vary with a different benchmark and evaluation measure. First, the artificial surfaces acquired from GlobeLand30-2010 (http://globallandcover.com/GLC30Download/index.aspx), a mapping product of global land cover at 30-meter spatial resolution derived from remote sensing images in 2010, was used as the built-up areas in the benchmark, see S3 File. Next, two evaluation measures, the similarity measure (*M*_*1*_), and an integrated measure (*M*_*2*_) averaging both correctness and completeness with equal weights, proposed in an existing study [1], were employed to compare the automatically delineated built-up areas and the corresponding benchmark.

As an example, “Table 9” lists the most-appropriate thresholds or ranges for 21 out of the 33 subdivisions of the North Island, using the GlobeLand30-2010 and the above evaluation measures, also see S4 File. The results of the other 12 subdivisions of the North Island are not listed here because the corresponding artificial surfaces for such a subdivision were not available in the GlobeLand30-2010.

**Table 9:** Most-appropriate thresholds or ranges for 21 out of the 33 subdivisions of the North Island, using the GlobeLand30-2010 and two different evaluation measures.

No. of Subdivision	Name of built-up areas	Size of built-up areas (km^2^)	Most-appropriate threshold or range					
			Street block size (km^2^)		Cell density (km/km^2^)		Kernel density (num/km^2^)	
			*M*_*1*_	*M*_*2*_	*M*_*1*_	*M*_*2*_	*M*_*1*_	*M*_*2*_
1	Auckland	435.024	1.0×1.0	1.0×1.0	5	5	17	15
2	Lower & Upper Hutt	43.785	0.8×0.8	0.8×0.8	7	6	27	23
3	Wellington	44.593	0.7×0.7	0.7×0.7	7	7	31	28
4	Napier	23.608	0.8×0.8	0.8×0.8	6	6	27	26
5	Porirua	26.852	0.9×0.9	0.9×0.9	6	6	21	19
6	Paraparaumu	25.308	0.9×0.9	0.9×0.9	5	5	21	21
7	Hastings	14.518	0.5×0.5	0.5×0.5	6	7	19	18
8	Whangaparaoa	19.015	1.2×1.2	1.2×1.2	6	5	18	14
9	Masterton	13.073	1.0×1.0	0.5×0.5	5	5	17	16
10	Havelock North	6.476	0.9×0.9	0.6×0.6	6	6	20	20
13	Orewa	3.257	0.5×0.5	0.5×0.5	6	4	26	26
14	Otaki	2.757	0.9×0.9–1.0×1.0	0.9×0.9–1.0×1.0	5	10	26	21
16	Flaxmere	4.436	0.8×0.8	0.6×0.6–0.7×0.7	5	6	22	22
17	Carterton	2.907	0.5×0.5	0.5×0.5	5	4	20	18
20	Snells Beach	2.238	0.4×0.4–0.7×0.7	0.4×0.4–0.7×0.7	6–7	6–7	20	17
21	Warkworth	3.529	1.2×1.2–+∞	1.2×1.2–+∞	6	6	17	17
22	Martinborough	1.301	0.4×0.4	0.4×0.4	8	6	25	25
23	Greytown	1.487	0.4×0.4–0.7×0.7	0.4×0.4–0.7×0.7	5	5	14	14
24	Featherston	1.766	0.3×0.3–0.4×0.4	0.3×0.3–0.4×0.4	7	7	26	26
25	Helensville	2.193	0.5×0.5	0.5×0.5	5	7	22	31
27	Wellsford	1.361	0.7×0.7	0.7×0.7	5	6	22	30

**Fig 8:**
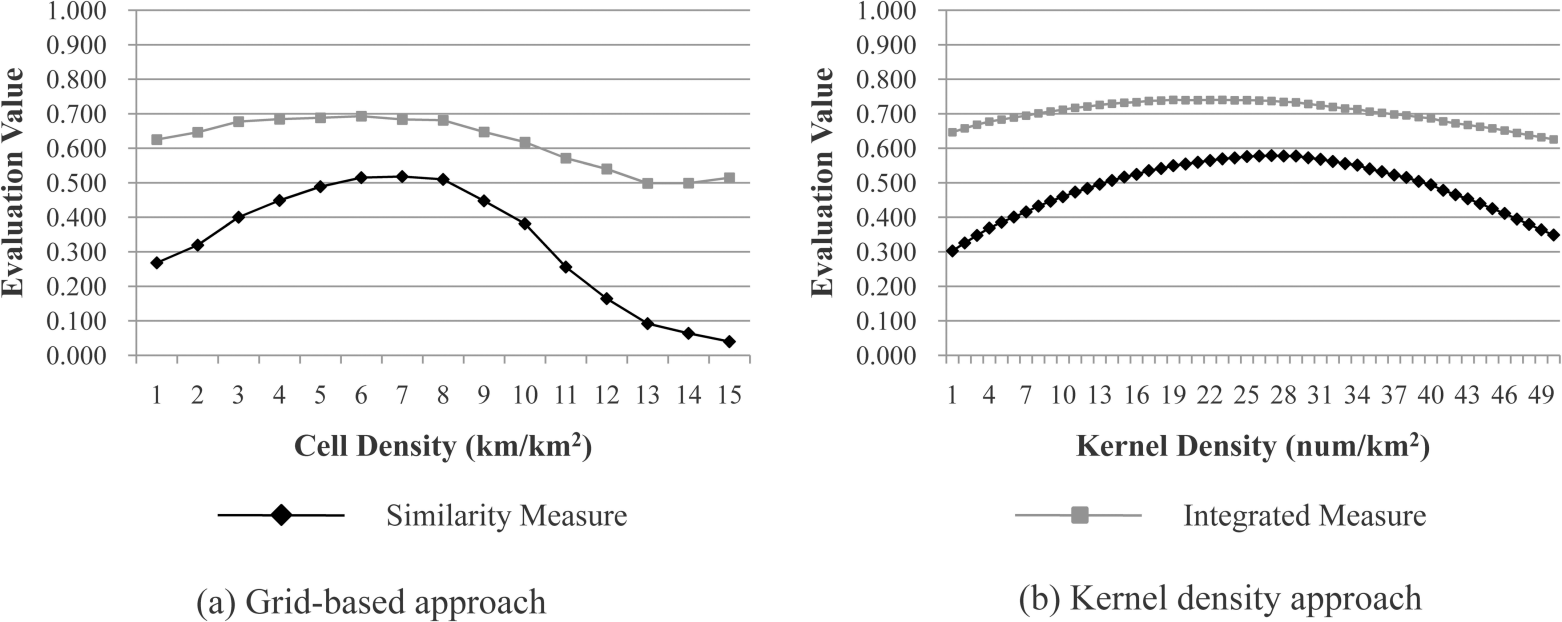
Evaluation of the thresholds for two parameters (cell density and kernel density) by using both the similarity measure (*M*_*1*_ and the integrated measure (*M*_*2*_, for the case study of Lower & Upper Hutt City.

The following can be observed from “Table 9”:

1. The appropriate threshold ranges are almost the same as those found in “Table 4”. For example, the most-appropriate thresholds or ranges for the street block size, cell density, and kernel density are mostly located within 0.4×0.4 to 1.0×1.0 km^2^, 5–7 km/km^2,^ and 17–28 num/km^2^, respectively; these ranges are almost the same as those found in “Fig 4” (0.4×0.4 to 0.9×0.9 km^2^, 5–7 km/km^2,^ and 18–31 num/km^2^) respectively. The majority of the most-appropriate thresholds or ranges are either are the same or overlap, even when using a different benchmark.
2. The majority of the most-appropriate thresholds or ranges are the same, irrespective of the two different evaluation measures (*M*_*1*_ and *M*_*2*_) used, which indicates the effectiveness of the similarity measure. Moreover, the similarity values are more sensitive to different parameter thresholds, whereas the integrated values are much closer to each other (“Fig 8”). If all the grid cells were divided into built-up areas, the accuracy would be very low, but the completeness would then be as high as 100%, and the integrated value would be larger than 50%. Thus, the similarity measure was selected to determine an appropriate threshold or range.

### Using a different study area

**Fig 9:**
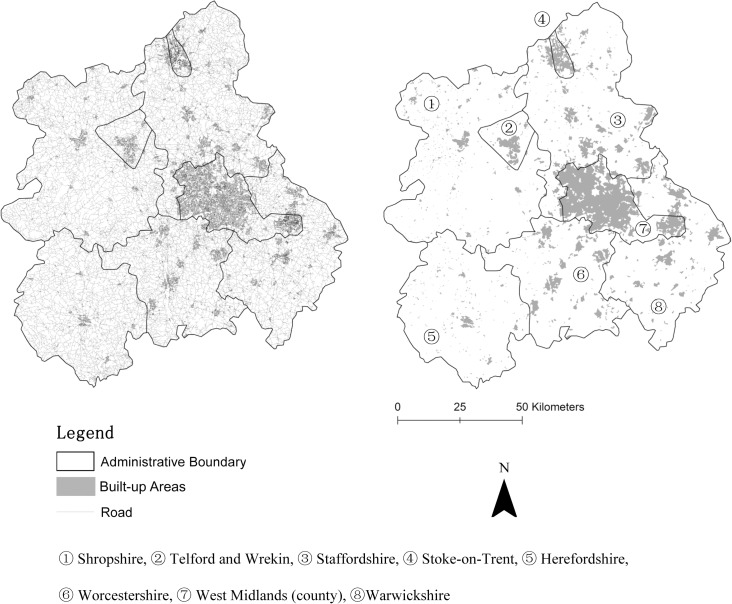
Study area of the West Midlands region of England.

The road network data of the West Midlands region of England (at 1: 25,000 scale) were also used for testing. This study area was chosen for three reasons. First, there are eight counties—Shropshire, Telford and Wrekin, Staffordshire, Stoke-on-Trent, Herefordshire, Worcestershire, West Midlands (county), Warwickshire—in the West Midlands region (see “Fig 9”) which makes a comparison of the most-appropriate thresholds for different counties possible. Second, the size of built-up areas varies with different counties. For instance, in both Stoke-on-Trent and West Midlands counties, most areas are built-up areas; but in other counties, most areas are non-built-up areas. Third and most importantly, the road network data of the West Midlands region were freely acquired from the open data provided by Ordnance Survey (https://www.ordnancesurvey.co.uk/). The corresponding artificial surfaces were also freely acquired from the GlobeLand30-2010 and were also used as the benchmarks, see S3 File.

**Fig 10:**
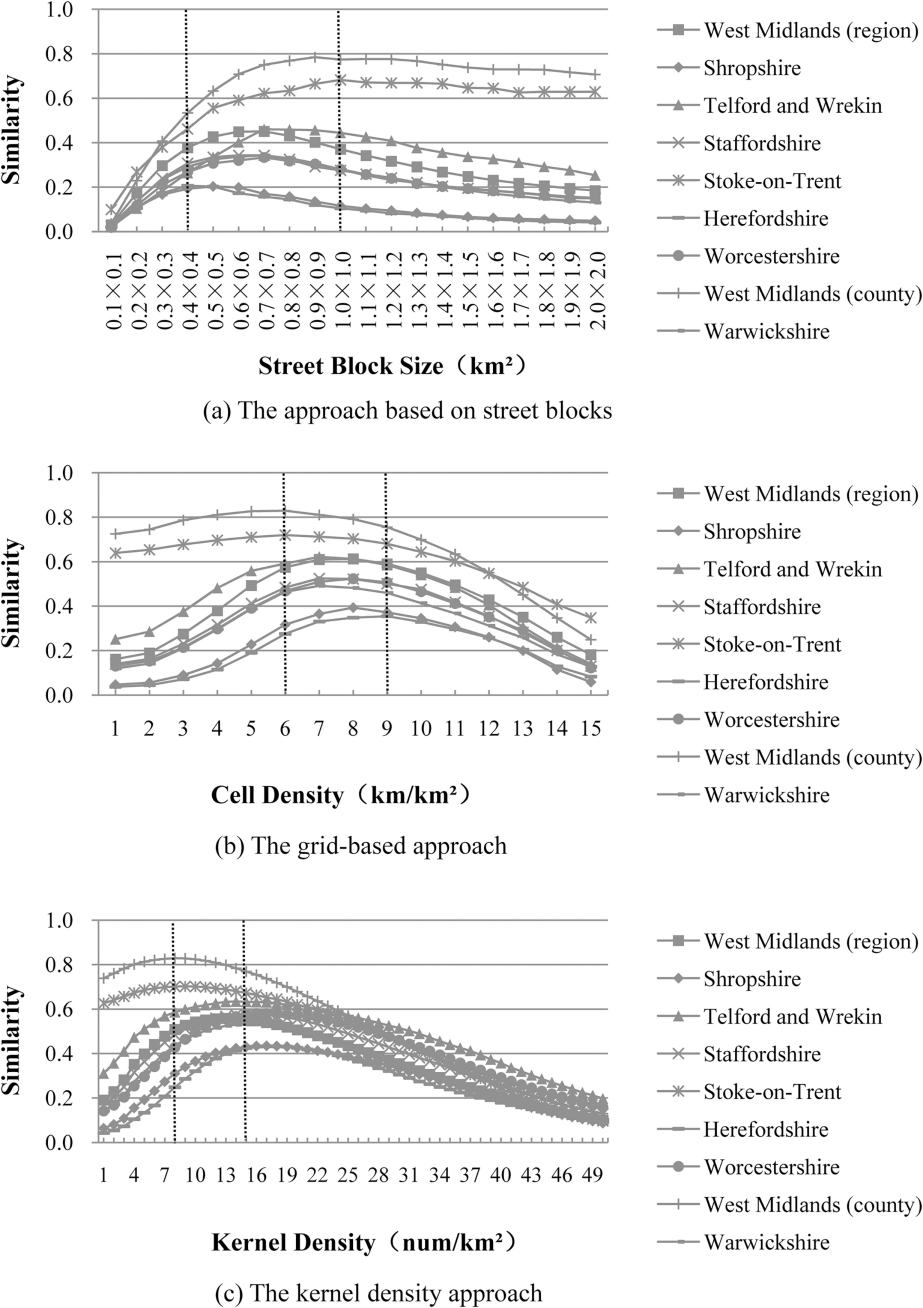
Most-appropriate thresholds for the West Midlands region of England and its eight counties using (a) the approach based on street blocks, (b) the grid-based approach, and (c) the kernel density approach.

“Fig 10” shows that the most-appropriate thresholds are located within 0.4×0.4 to 1.0×1.0 km^2^, 6–9 km/km^2^ respectively using the approach based on street blocks and the grid-based approach; these ranges are almost the same as those found before (“Fig 4” and “Table 9”). For the kernel density approach, seven out of the nine, most-appropriate thresholds (located within 13–15 num/km^2^) are very close to each other. Although the most-appropriate thresholds for West Midlands county and Stoke-on-Trent county were much smaller (e.g., 8 and 9 num/km^2^, respectively), the most-appropriate threshold ranges for these two counties (e.g., 3–14 and 3–17 num/km^2^, respectively) still overlap with those of other counties. However, the similarity distributions for both West Midlands county and Stoke-on-Trent county are different from those for other counties because most areas in these two counties are built-up. Even when all the street blocks or grid cells were delineated as built-up areas, the corresponding similarity values were still higher than 0.6 (“Fig 10”).

Furthermore, the artificial surfaces in the GlobeLand30-2010 suffer from classification errors. The overall accuracy of GlobeLand30-2010 (http://www.globeland30.org/home/Enbackground.aspx) was reported as 80.33%. Therefore, some small cities or towns were not used for comparison. Although the use of other source data (e.g., census data and “check-in” data) may offer alternatives, they also have limitations. For instances, check-in data often contains biases because not everyone checks in or uses social media. As it is difficult to precisely identify built-up areas, we suggest using different source data as benchmarks in order to minimize subjectivity.

## Conclusion and discussions

This study employed an empirical approach to determine appropriate thresholds for multiple parameters in the delineation of built-up areas using road network data. Specifically, the five parameters (street block size, cell size, cell density, bandwidth and kernel density) of the three typical approaches (the approach based on street blocks, the grid-based approach and the kernel density approach) were tested. That is, extensive experiments were carried out to investigate the most-appropriate thresholds for various parameters in these approaches. The North Island of New Zealand was chosen as the study area, with road network data used as source data, and the corresponding building and residential data used as benchmarks. The road network was divided into nine administrative subdivisions and 33 different city/town subdivisions. The built-up areas of each subdivision were automatically delineated with different approaches and thresholds. For each subdivision, the most-appropriate threshold was determined by calculating the similarity (or consistency) between an automatically delineated built-up area and a corresponding benchmark. A different benchmark (GlobeLand30-2010), an integrated measure, and a different study area (the West Midlands region of England) were used for validation of earlier results. Results show that in most cases, the most-appropriate thresholds for the different subdivisions were either the same or close to each other. However, the most-appropriate thresholds for some cities/towns varied dramatically.

**Fig 11:**
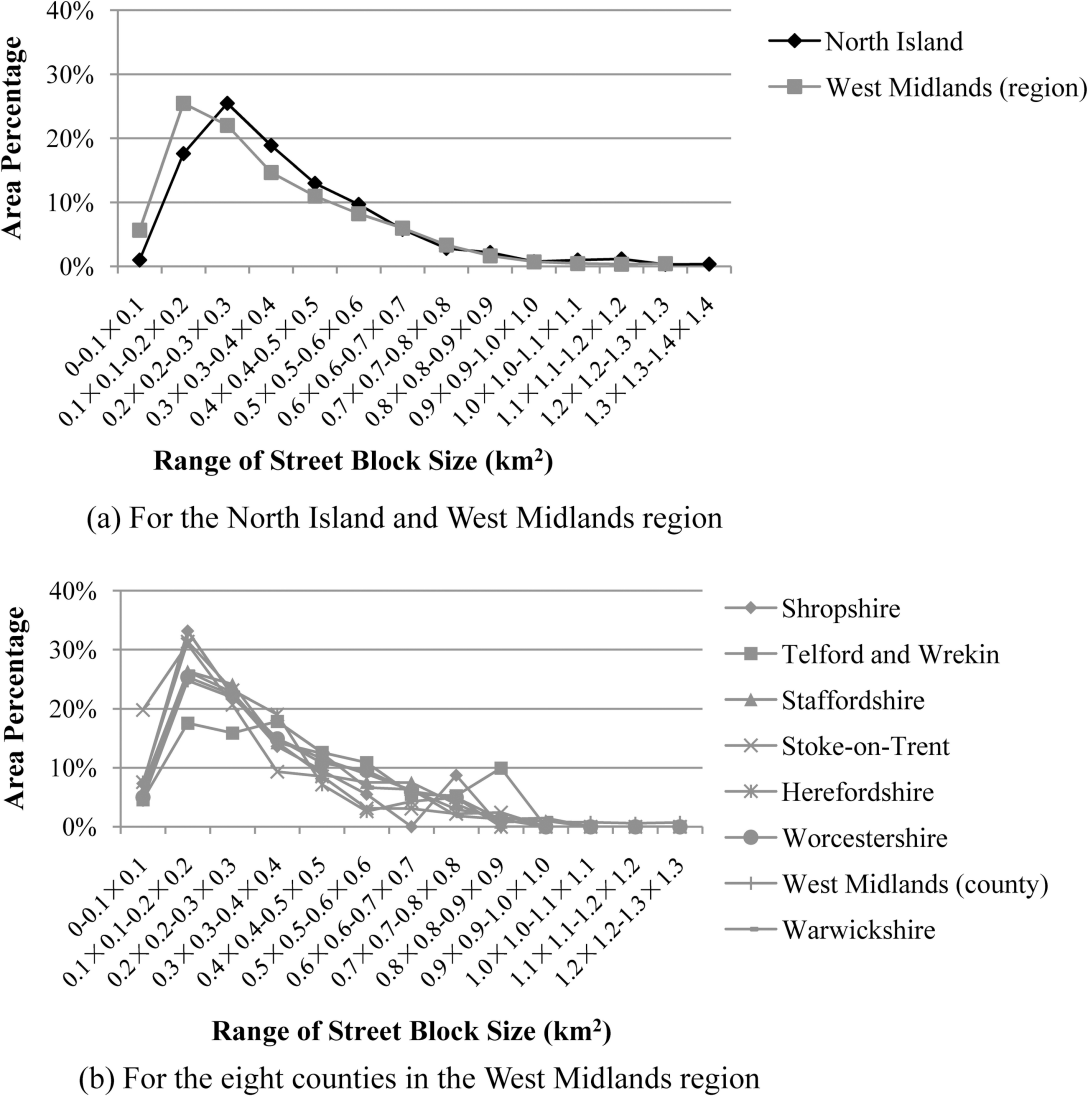
Plot of the area percentages of street blocks in the built-up areas of both the North Island and West Midlands region (a) and eight counties in the West Midlands region (b). The x-axis denotes the different area ranges of street blocks in the built-up areas and the y-axis denotes the total area of street blocks within an area range proportional to that of street blocks within all the (area) ranges.

**Fig 12:**
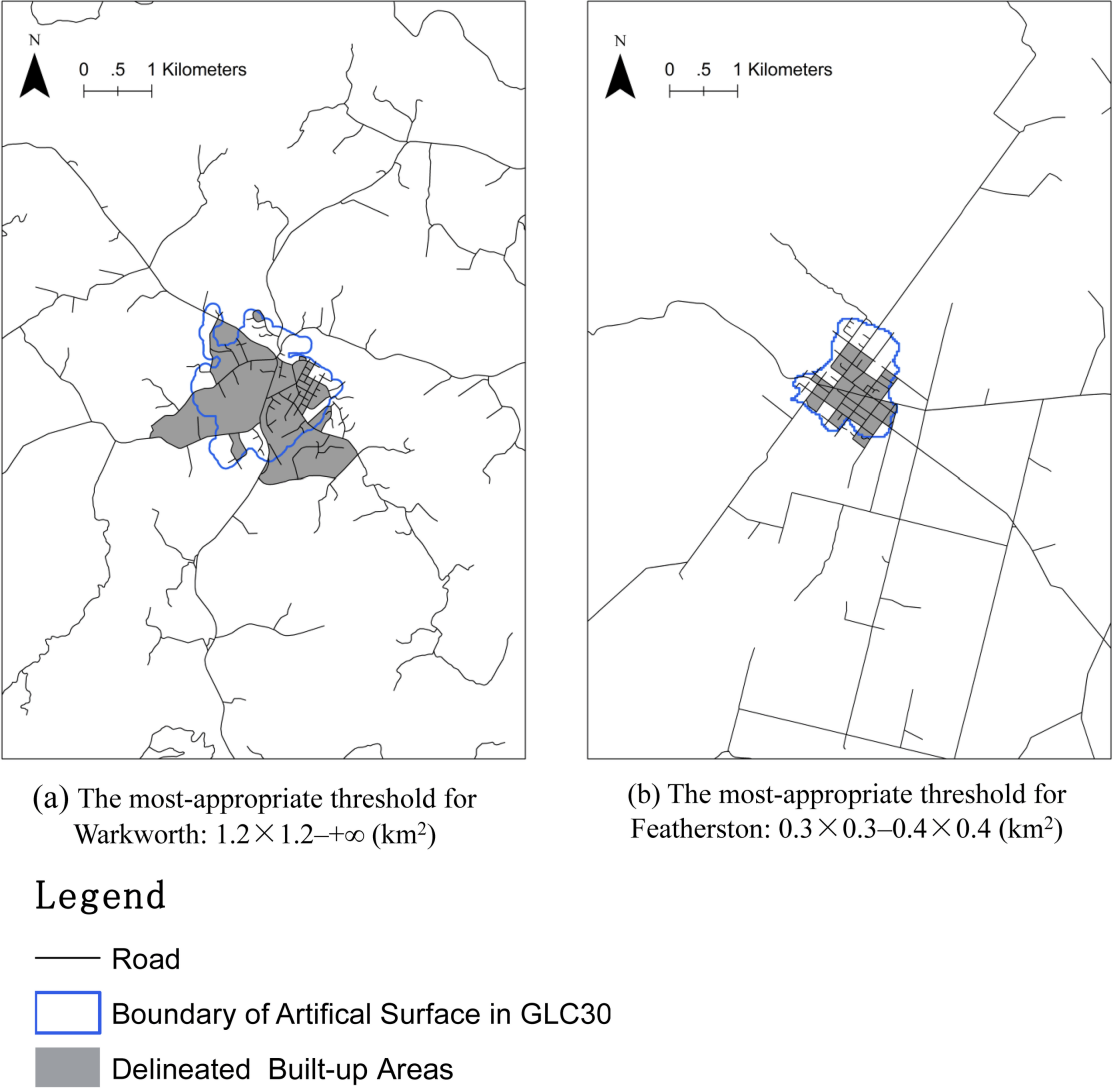
The most-appropriate thresholds for two small towns (Warkworth and Featherston) in the North Island.

The reasons for these results might be as follows:

1. The road network of a city/town is commonly designed based on principles and criteria proposed by the department of urban planning. Consequently, the street block size or cell density of a road network in a city/town region are organized so as to be not to be too large or too small. For instance, 95% of the street blocks in the built-up areas of either the West Midlands region or North Island are smaller than 0.9×0.9 km^2^ (“Fig 11A”). Such principles and criteria may be consistent within a country (“Fig 11A”) and even be similar for different countries (“Fig 11B”). Most of the appropriate thresholds are therefore the same or similar.
2. However, different cities/towns often have different street network patterns (e.g. street blocks of different sizes and shapes), so the most-appropriate thresholds are not always the same and may even be quite different (“Fig 12”).

Nevertheless, this study validates that the empirical approach [26] is also applicable to the delineation of built-up areas. That is, to first subdivide a large road network according to administrative boundaries, or smaller units (e.g., cities or towns), and then to apply the most-appropriate thresholds or ranges obtained from multiple subdivisions to infer the results for the larger one. The inference method may include calculating the average or median of multiple, most-appropriate thresholds, or overlapping multiple appropriate threshold ranges [26].

In future research, more road network data of different countries and/or regions will be used for testing the most-appropriate thresholds for various parameters. In addition, other source data (e.g. census data) will be used as benchmarks for automatic analysis of delineated built-up areas. Moreover, it will still be necessary to develop an approach to adaptively determine the most-appropriate threshold for different cities/towns. Finally, it may be worth investigating whether the empirical approach is also applicable for determining appropriate thresholds for the delineation of built-up areas with different source data.

## Supporting information

S1 File Road, buildings and residential data provided by the Land Information of New Zealand (https://data.linz.govt.nz/). (ZIP)

S2 File The results for the 33 subdivisions of the North Island using the similarity measure. (ODS)

S3 File Land cover data provided by the National Geomatics Center of China (http://globallandcover.com/User/Login.aspx). (ZIP)

S4 File The results for the 21 out of the 33 subdivisions of the North Island using both the similarity and the integrated measures. (ODS)
